# The effect of job satisfaction regulating workload on miners’ unsafe state

**DOI:** 10.1038/s41598-022-20673-y

**Published:** 2022-09-30

**Authors:** Lei Chen, Hongxia Li, Lin Zhao, Fangyuan Tian, Shuicheng Tian, Jiang Shao

**Affiliations:** 1 College of Safety Science and Engineering, Xi’an University of Science and Technology, Xi’an, 710054 China; 2 Institute of Safety and Emergency Management, Xi’an University of Science and Technology, Xi’an, 710054 China; 3 School of Management, Xi’an University of Science and Technology, Xi’an, 710054 China; 4 School of Management, Henan Institute of Urban Construction, Pingdingshan, 467000 China; 5 School of Architecture & Design, China University of Mining and Technology, Xuzhou, 221116 China

**Keywords:** Statistics, Engineering, Risk factors

## Abstract

Miners’ unsafe behavior is the main cause of accidents in coal mines, and unsafe state have an important influence on unsafe behavior among miners. To minimize accidents from the source of accident chain, we evaluated the impact of workload on miners’ unsafe state. It is important for coal enterprises to monitor miners’ unsafe state and to prevent unsafe accidents. Workload is divided into two dimensions: work time and work demand. Meanwhile, we introduced job satisfaction as a moderating variable. Through empirical research methods, first-line employees from two coal mines in China were enrolled in the questionnaire survey. Regression analysis was used to verify the impact of workload and its various dimensions, job satisfaction, and miners’ unsafe state. We found that workload, work time and work demand have significant positive effects on miners’ unsafe state. Job satisfaction plays a moderating effect in the relationship between workload and miners’ unsafe state. To some extent, a higher job satisfaction was associated with reduced workload, reduced occurrence of miners’ unsafe state and minimal incidences of unsafe accidents. On this basis, measures were proposed to improve miners’ unsafe state in terms of workload and job satisfaction. This study informs the establishment of effective intervention measures to monitor miners’ unsafe state and is also beneficial to the improvement of coal mine safety.

## Introduction

In recent years, work safety and its associated health risks have become the focus of attention in industrial development^1^. This is especially true for the coal industry, which has the most accidents in industrial production. It has been reported that coal will continue to be the dominant energy source in China for a foreseeable future. As an important pillar in China’s economy, the coal industry guarantees social production and life^2,3^. The coal mine production environment is a high-risk area that is prone to disasters such as coal dust and gas bursts^4,5^. Compared to other industries, coal miners work in more dangerous environments^6^. There are many people engaged in this industry in China, which is a special professional group^7^. With advances in science and technology as well as the improvement of coal mine safety management systems, the safety situation of coal mine production in China has steadily improved, yet coal mine accidents still occur^8,9^. The occurrence of mining disasters do not guarantee the safety of coal mine workers^10^. To minimize the occurrence of coal mine accidents, it is necessary to intervene in the process of coal mining through technology and management to predict accidents in advance^11^. To prevent safety associated accidents during coal mining, it is necessary to evaluate the factors and mechanisms that lead to coal mine safety accidents. Human error is an important cause of unsafe accidents, and human state is a key factor that affects behavioral choices^12^. Therefore, from the perspective of human factors, it is necessary to evaluate the safety of a miner’s state and the mechanism of unsafe state during the current severe situation of coal mine production.

Studies have shown that unsafe behaviors directly caused by workers’ state are the main reason for accidents. For example, the probability of minor non fatal accidents in wake-up in the morning and heavy workload without resuming sleep is higher than that in normal work and leisure^13^. The health state of miners is related to the risk of coal mine accidents^14^. The well-known tool for analyzing human factors of accidents (human factors analysis and classification system) lists the operator’s state as a prerequisite for unsafe behavior. Chen et al.^15^ reported that operator condition is a category in the precondition levels associated with unsafe behavior. Miners’ unsafe behavior is dominated by their unsafe state. The main causes of unsafe behaviors are fatigue and poor mental state of coal mine operators^16^. Long term mental fatigue, poor motivation, low vigilance and inattention will have a serious impact on people’s brain cognition, which will be manifested as a sub-health state, and then turn into an unsafe state^17^. The accumulation of unsafe state will affect people’s brain to make wrong behavior decisions. At present, there are many devices that can monitor the physiological indexes of people to judge their state. When physiological indicators of employees (especially front-line workers) do not meet safety state standards, they should be advised to take corresponding measures to prevent accident^18^. For example, early warning through communication, or take certain measures to urge them to improve the unsafe state. In some cases, workers should not engage in high-risk work for long periods. If necessary, real-time monitoring and safety training should be performed to reduce the occurrence of unsafe behaviors.

Human unsafe state, that is, a safe state or a dangerous state under the combination of time and space attributes, plays a subjective role in the accident chain. Wang et al.^19^ reported that operator state is the ability to complete current tasks under the influence of internal states, including emotional state and fatigue. In their study on safety state and analysis of maintenance personnel, Bi et al.^20^ defined safety state of human beings as the ability to complete a specified work within time and accurately under the specified time and actual working conditions. To sum up, combined with the actual situation and working characteristics of miners, this paper defines the miners' unsafe state as a scenario that may lead to unsafe behaviors of miners under the joint action of time and space in a specific environment. This unsafe state is affected by physiology, psychology and knowledge and so on. Functional state information such as physiological and psychological signals generated by different workloads can influence the reallocation of operator state tasks^21^. Feng et al.^22^ concluded that workload has a significant impact on unsafe behavior in coal mines. Workload governs behavioral decisions by influencing the emotional state of miners. Cognitive workload assessment plays a critical role in the mental safety of the brain during emergencies. It can also be used for brain disease diagnosis and monitoring of mental health state^23^. The high workload of mental work will reduce the work efficiency of employees, and in severe cases will lead to physical and psychological insecurity. Reasonable workload can effectively improve work efficiency and save human resources^24^. Workload can affect the alertness of employees working in harsh environment and the ability to complete multiple tasks and compete for limited attention resources. By monitoring the safety state of the operator, it is possible to reduce the possibility of serious errors and provide ergonomic information related to performing tasks. Zhou et al.^25^ studied the role of job satisfaction in the impact of psychological load on job performance. The results show that psychological load has a direct negative impact on job performance through job satisfaction. The research shows that the greater the workload, the lower the job satisfaction of employees^26^. Job knowledge state, such as work-related resources and stressors, can affect employee job satisfaction. By monitoring occupational health and safety, it can facilitate the assessment and improvement of the safety state of people in the workplace^27^. Employee’s job satisfaction can change their states, such as depression, psychological expectations can not reach the expected value. Decreased operator performance due to minimal vigilance, fatigue, and job dissatisfaction can easily lead to unsafe behaviors, which are the main causes of some serious disasters^28^. When operators suffer from sleep deprivation, depression or fatigue due to irrational work, it leads to a decline in operational ability, thereby seriously affecting work efficiency and safety^29^. A high-pressure work has a negative impact on people’s emotions, which is easy to produce an unsafe state^30^.

The production process of coal is complex system, and the behavior of miners is closely associated with the operation process, which directly determines the safety of the whole production process^31^. Therefore, unsafe states, which are the source of miners’ unsafe behaviors, should be monitored and corresponding safety measures put in place, so as to reduce unsafe behaviors and prevent accidents. From the miners’ perspective, we selected workload and job satisfaction as indicators to study their impact on miners’ unsafe state, so as to provide basis for reducing miners’ unsafe behavior.

## Literature review and hypotheses

### Workload, its dimensions and miners’ unsafe state

Workload is a kind of pressure source that is based on job requirements, which has attracted much attention in the field of organization management in recent years. Veltman et al.^32^ reported that workload is the cost employees have to pay to complete tasks, including subjective cognition of physical and psychological input. Kirmeyer et al.^33^ reported that workload is the amount of work that an individual bears per unit time. Wickens^34^ reported that workload is reflected in individual work demand load. Maslach et al.^35^ divided workload into two kinds: quantification of work requirements and quality of work requirements. Generally, in research, quantification of work requirements is emphasized to explain workload. Caplan et al.^36^ defined workload as a comprehensive sense of self-efficacy when an individual performs a special task or multiple tasks in a given environment.

Workload is not conducive for maintaining a good state of safety for employees. According to the theory of self-control resources, individual’s control of resources is limited. When an individual’s control of resources exceed their own ability and cannot be supplemented in time, it is easy to have a bad state, resulting in an inability to effectively control their own behavior. High workload often lead to reduced self-control resources for miners, which reduces their ability to maintain individual behavior in accordance with enterprise norms, resulting in unsafe state^37^. Chang et al.^38^ reported that subjective fatigue load affects work safety state of employees. Xing et al.^39^ reported that miners in unsafe state of fatigue increased the possibility of accidents. Reducing workload and fatigue is of great significance to prevent accidents.

Based on the requirements of quantity and quality in workload definition, we elaborated workload from two dimensions of work time and work demand. Work time is the number of hours an employee has worked on the job, which mainly refers to work length. Work demand is the physical and psychological (cognitive, emotional) inputs required by employees to complete a certain job, which mainly refers to work intensity^40^. Since work time and work demand are two dimensions for measuring workload quality and quantity, their impact on miners’ unsafe state may be different.

Ng et al.^41^ used the social identity theory to evaluate the impact of work time on employees’ identity. They found that an increase in work time reduces employees’ identity of occupation and organization, and makes them lack a sense of security at work. Haines et al.^42^ studied 7802 full-time paid employees from a psychological perspective. They found that longer work time increases negative emotions and psychological pressures for employees, which leads to unsafe states such as anxiety and fatigue. Hulst^43^ reported that longer work time leads to fatigue and frequency of unhealthy behaviors (drinking and smoking), and may even lead to various diseases. Work demand consume psychological resources that are used to deal with psychological needs, and resource exhaustion leads to unsafe states such as employee fatigue and uneasiness. McEwen^44^ linked that socio-psychological pressure and complaints, job burnout, fatigue, dissatisfaction with current work situation and other factors are closely associated with workload, which can cause adverse physiological reactions, such as increased blood pressure, heart rate and other unsafe states. Therefore, we proposed the following hypotheses.

#### Hypothesis 1 (H1)

Workload has a significant positive effect on miners’ unsafe state.

#### Hypothesis 1a (H1a)

Work time has a significant positive effect on miners’ unsafe state.

#### Hypothesis 1b (H1b)

 Work demand has a significant positive effect on miners’ unsafe state.

### The moderating effect of job satisfaction

Job satisfaction is an individual’s psychological response to the work they are engaged in. Weiss^45^ reported that job satisfaction is the employees’ evaluation of their work, which reflects employee recognition of things. If employees regard their work as a career, they will be in a positive and high pitched state, and will pay more time costs. Job satisfaction can improve job performance, while reduced job satisfaction can lead to unsafe behavior. Higher job satisfaction can keep employees in good state, and implementation of a plan to improve employee satisfaction can lead to a reduction in occupational accidents^46^. The traditional view is that job satisfaction is a kind of work attitude, which is mainly reflected in personal overall work evaluation, and personal work cognition affects work attitude^47^. When employees feel a higher level of job satisfaction, and their work returns meet their psychological expectations, they will have a positive work attitude. On the contrary, employees are prone to complain, work slack and other emotions, which can easily lead to unsafe state and behavior, thereby affecting work progress. By evaluating the relationships among psychological capital, job satisfaction and organizational citizenship behavior, Jung et al.^48^ concluded that employee expectation and optimistic attitudes have a positive impact on job satisfaction.

Chen et al.^49^ evaluated 622 grassroots medical staff in four sample cities and found that job satisfaction has a moderating effect on fatigue and job burnout. Various studies have divided job satisfaction into multiple dimensions, including welfare benefits, performance appraisal, and organizational safety management. A high job satisfaction for employees is an appropriate balance between amount of work time and work demand, and reduced workload. With a high sense of job satisfaction, employees have a stronger sense of work belonging, are more involved in their work, and have reduced insecurities^50^. Improved job satisfaction levels are associated with reduced workload and unsafe psychology, enhanced safe state for employees at work, thereby reducing employees’ anti production behavior. Therefore, we proposed the following hypothesis.

#### Hypothesis 2 (H2)

Job satisfaction plays a moderating effect between workload and miners’ unsafe state.

## Methodology and research design

Using a combination of quantitative and qualitative methods, this study collected data on 385 front-line coal miners randomly sampled from two coal mines in China. Then, interviews were conducted with relevant experts in the field for the measurement items, so that the research items fit the actual situation of coal mine safety production. After an extensive review of the literature to identify study variables and measure items, we compiled a completed questionnaire. We use structural equation model to formally evaluate the relationship between three variables, namely, workload, job satisfaction and miners’ unsafe state. SPSS 19.0 and AMOS 22.0 were used for statistical analyses in this paper.

### Variable measurements

Based on existing research basis at home and abroad, drawing on the mature scale of previous studies, combined with surveys of coal miners, a questionnaire was developed. A five-point Likert scale was used in the questionnaire, and each score represented the extent to which the respondents agreed with the presented statement.

### Workload scale

Workload in this study was measured from two dimensions of work time and work demand. The specific measurement methods were: i. Work time: Measurement items were compiled with reference to the results of previous studies^51,52^. Work time was measured by the number of hours per week. There are two items. For example, “How many hours did the employee work per week in the past month?”. ii. Work demand: Measurement items were compiled with reference to the results of previous studies^53,54^. A scale with six items was designed. For example, “To what extent do operating posts require hard work?”.

Based on the above analysis, a workload measurement scale was formed, consisting of two dimensions: work time and work demand, with a total of 8 items.

### Job satisfaction scale

Currently, measurement of job satisfaction was mostly multi-dimensional. There were several kinds of job satisfaction measurement questionnaires commonly used by domestic and foreign scholars. (i) Minnesota Satisfaction Scale. This scale was compiled by Weirs et al. It is a relatively mature job satisfaction measurement scale, but the related items are not very perfect. (ii) Job description index scale. This scale was compiled by Smith et al. Measurement results of the scale are accurate and universal. The disadvantage of this scale is that it pays too much attention to the main content, and the measurement content does not include all factors. (iii) Domestic employee satisfaction scale. This scale was developed by a Chinese scholar Ling Wencuo and others with reference to the Western research foundation and the domestic situation. In practice, the scale has shown good reliability-validity. iv. Job satisfaction index scale. This scale was compiled by Brayfield et al.^55^ A total of six items are included, covering aspects such as leaders, colleagues and personal work itself. The questionnaire has been cited and verified in research, and is a very mature scale.

Due to the high-intensity work and overall education level of miners, the questionnaire should have less questions that are simple and easy to understand. We revised the job satisfaction scale to 6 items.

### Miners’ unsafe state scale

In previous studies on accidents, unsafe behavior of humans and unsafe state of things were evaluated, while unsafe state of people were ignored. He^56^ reported that human unsafe state is the biggest safety hazard. Studies on miners’ unsafe state are relatively less. Based on the analysis of coal mine accidents, combined with views of relevant scholars, we compiled the measurement scale of miners’ unsafe state from the aspects of physiology, psychology, safety awareness and habitual wrong behavior. After reliability-validity analysis, our results can guide coal mining enterprises’ safety management of miners. There were 8 items in total.

An initial questionnaire was formed, the expression of each item in the questionnaire was accurate, clear, and easy to understand. Questionnaire items were repeatedly discussed and revised. Finally, relevant experts were contacted to review the questionnaire and confirm that its title reflects the actual situation of the enterprise. To test the reliability-validity of the questionnaire, a small-scale pilot study was performed. After pre-investigation, the questionnaire was analyzed and tested for reliability-validity, which was the basis for preparation of a formal questionnaire. If there is an item that contributes less to the overall scale than the contribution of all items to the overall scale, it is deleted^57^. From the pre-investigation, the questionnaire was further simplified and modified, and the final scale was determined for this study. The formal questionnaire was shown in Table 1.

**Table 1:** Questionnaire.

Variable	Dimension	Item	Measurement item
Workload	Work time	*I*_*1*_	To what extent can I determine my daily work time based on my work schedule?
		*I*_*2*_	To what extent can I meet my physiological requirements every day, except for work?
	Work demand	*I*_*3*_	To what extent do your current jobs require you to work hard?
		*I*_*4*_	To what extent do you have a lot of tasks to do in your current job?
		*I*_*5*_	To what extent do you have insufficient time to do your work?
		*I*_*6*_	To what extent do you have excess tasks to fulfill in your current job?
		*I*_*7*_	To what extent do you think there is not enough time to complete the work?
		*I*_*8*_	To what extent do you face conflicts from a variety of different job requirements?
Job satisfaction	–	*I*_*9*_	I am satisfied with the nature of the work I am doing
		*I*_*10*_	I am satisfied with my superiors (who directly instructed me to work)
		*I*_*11*_	I am satisfied with the relationship with the workers I work with
		*I*_*12*_	I am satisfied with my work income
		*I*_*13*_	I am satisfied with the promotion opportunities I can get at work
		*I*_*14*_	Considering every aspect of my work, I am satisfied with my current work situation
Miners’ unsafe state	–	*I*_*15*_	I can't work normally because of the bad environment (climate parameter) underground
		*I*_*16*_	When the body is tired or sick, I will keep working
		*I*_*17*_	I sometimes start working with emotions (affected by family relationships, death of relatives, etc.)
		*I*_*18*_	Before work, I occasionally use drinking to relieve the unhappiness at work
		*I*_*19*_	I think the relevant safety professional knowledge is enough to solve the problems at work
		*I*_*20*_	At work, I sometimes try to save trouble and do not strictly abide by the safety rules and regulations
		*I*_*21*_	At work, I sometimes maintain the safety protection device according to my own habits
		*I*_*22*_	At work, I sometimes use my hands instead of prescribed tools to operate

### Ethical approval and consent to participate.

In 2021, we surveyed front-line workers at Hongliulin Coal Mine of Shaanxi Coal Group and Lugou Coal Mine of Zheng Coal Group in China. This project was approved by the Institutional Review Board of Xi'an University of Science and Technology. All methods were carried out in accordance with relevant guidelines and regulations. Written informed consent was obtained from each subject before enrollment. All subjects provided written informed consent prior to being monitored.

## Data collection and analysis

### Questionnaire distribution and data collection

According to the division of work types, a random sampling method was used to investigate the front-line coal miners of two coal mines in China. A total of 385 questionnaires were distributed from which 351 were collected. After eliminating invalid questionnaires, 307 valid questionnaires were obtained. Effective questionnaire recovery rate was 87.5%. SPSS 19.0 was used to perform descriptive statistical analysis of samples. Study participants were from all age groups. Among them, the 31–40-year-old group was the most represented, accounting for 33.88% of the effective survey population. There were more married participants than unmarried participants. From education level perspective and characteristics of front-line employees in coal mines, high school group accounted for the largest proportion, accounting for 58.31% of the effective survey population. From the working age perspective, it covers all age groups. The 5–10-year group accounted for 34.2% of the study population. The demographic information obtained from this survey is consistent with the actual situation of coal enterprises and has statistical significance.

### Reliability analysis

The SPSS19.0 was used to test the consistency of the Cronbach’s α coefficient of the questionnaire. The workload scale was 0.793, job satisfaction scale was 0.839, miners’ unsafe state scale was 0.778, and each scale was greater than 0.7. It shows that reliability of the scale was high, and analysis can be continued.

### Validity analysis

Firstly, the kmo values of workload, job satisfaction and miners’ unsafe state were 0.804, 0.859 and 0.827 respectively. The sig. values of Bartlett's spherical test were all 0.000, indicating that it was suitable for factor analysis.

Then, principal component analysis and total variance explanation table were used to analyze the variables.The results were shown in Table 2.

**Table 2:** Validity test results.

Variable	Item	Factor load	Proportion of explained variance (%)
Workload	*I*_1_	0.816	60.037
	*I*_2_	0.812	
	*I*_3_	0.811	
	*I*_4_	0.810	
	*I*_5_	0.809	
	*I*_6_	0.805	
	*I*_7_	0.804	
	*I*_8_	0.802	
Job satisfaction	*I*_9_	0.812	55.614
	*I*_10_	0.816	
	*I*_11_	0.814	
	*I*_12_	0.810	
	*I*_13_	0.815	
	*I*_14_	0.812	
Miners’ unsafe state	*I*_15_	0.802	63.706
	*I*_16_	0.804	
	*I*_17_	0.800	
	*I*_18_	0.801	
	*I*_19_	0.807	
	*I*_20_	0.800	
	*I*_21_	0.801	
	*I*_22_	0.799	

Confirmatory factor analysis was used to verify the convergent and discriminant validity of the scale. The results were shown in Tables 3 and 4.

**Table 3:** Model AVE and CR metrics results.

Variable	Average variance extracted(AVE) value	Combined reliability(CR) value
Workload	0.567	0.805
Job satisfaction	0.669	0.867
Miners’ unsafe state	0.518	0.777

**Table 4:** Discriminant validity: Pearson correlation and AVE square root value.

Variable	Workload	Job satisfaction	Miners’ unsafe state
Workload	0.606	–	–
Job satisfaction	− 0.138	0.685	-
Miners’ unsafe state	0.087	− 0.036	0.564

Average Extraction of Variance (AVE) and Combined Reliability (CR) were used for convergent validity analysis. Usually, the AVE is greater than 0.5 and the CR value is greater than 0.7, indicating that the convergent validity is high. It can be seen from Tables 2 and 3 that the factor load of each item was greater than 0.5, the corresponding CR value of the three factors was greater than 0.7, and the AVE value was greater than 0.5, indicating that the scale had good convergent validity. It can reasonably explain the three concepts of workload, job satisfaction and miners' unsafe state.

Confirmatory factor analysis (CFA) can be used to study discrimination validity. If the square root value of the AVE of a factor is greater than the absolute value of the correlation coefficient between the factor and other factors, and all factors show such a conclusion, it indicates that it has good discrimination validity. It can be seen from Table 4 that the AVE square root value of the workload was 0.606, which was greater than the maximum value of the absolute value of the inter-factor correlation coefficient of 0.138, indicating that it had good discrimination validity. The AVE square root value of job satisfaction was 0.685, which was greater than the maximum value of the absolute value of the correlation coefficient between factors of 0.138, indicating that it had good discrimination validity. The AVE square root value of the miner’s unsafe state was 0.564, which was greater than the maximum value of the absolute value of the correlation coefficient between the factors of 0.087, indicating that it had good discrimination validity.

Amos 22.0 was used for confirmatory analysis of each measurement model of the scale. The specific results were shown in Table 5.

**Table 5:** Model fitting results.

Index	Absolute fit			Value-added fit		
	χ^2^/df	RMSEA	GFI	NFI	IFI	CFI
Fitting results	3.927	0.047	0.852	0.917	0.928	0.912
Fitting standard	3–5	< 0.05	> 0.80	> 0.90	> 0.90	> 0.90
Validation results	Acceptable	Better fit	Acceptable	Good fit	Good fit	Good fit

χ^2^/df represents the ratio of chi-square to degrees of freedom. RMSEA represents the root mean square error of approximation. GFI represents fitness index. NFI represents normed fit index. CFI represents comparative fit index.

Table 5 shows that the calculation results of each index of the relationship model of workload, job satisfaction and miners’ unsafe state are in the acceptable range from the goodness of fit. It shows that the model meets the requirements.

## Model verification and result analysis

### Correlation analysis of workload and its dimensions and miners’ unsafe state

To evaluate the correlation between independent variables and dependent variables, statistical analysis and tests were performed on these variables. Pearson correlation analysis was used to test the correlation between workload and its dimensions and miners’ unsafe state. The analysis results were shown in Table 6.

**Table 6:** Correlation analysis results of workload and its dimensions and miners’ unsafe state.

Variable	Miners’ unsafe state		
	Pearson correlation	P	N
Workload	0.587*	0.019	307
Work time	0.118*	0.039	307
Work demand	0.560**	0.000	307

p represents the significance value. *represents a significant level at p < 0.05. **represents a significant level at p < 0.01. N represents the sample size.

The correlation coefficient between workload and miners’ unsafe state was 0.587 (p = 0.019 < 0.05), indicating that there was a positive correlation between workload and miners’ unsafe state. Correlation coefficient between work time and miners’ unsafe state was 0.118 (p = 0.039 < 0.05), indicating that there was a positive correlation between work time and miners’ unsafe state. Correlation coefficient between work demand and miners’ unsafe state was 0.560, at a significant of 0.01 level, indicating that there was a significant positive correlation between work demand and miners’ unsafe state.

### Hypothesis test

From the above analysis of the correlation between independent variables and dependent variables, there was a significant correlation between workload and each dimension and miners’ unsafe state. Correlation analysis reflects the degree of correlation between variables, and can not prove causal relationships between variables. Therefore, on the basis of the above correlation analysis, ridge regression method was used to test the research hypothesis of this study.

### Impact of workload and its dimensions on miners’ unsafe state

**Figure 1:**
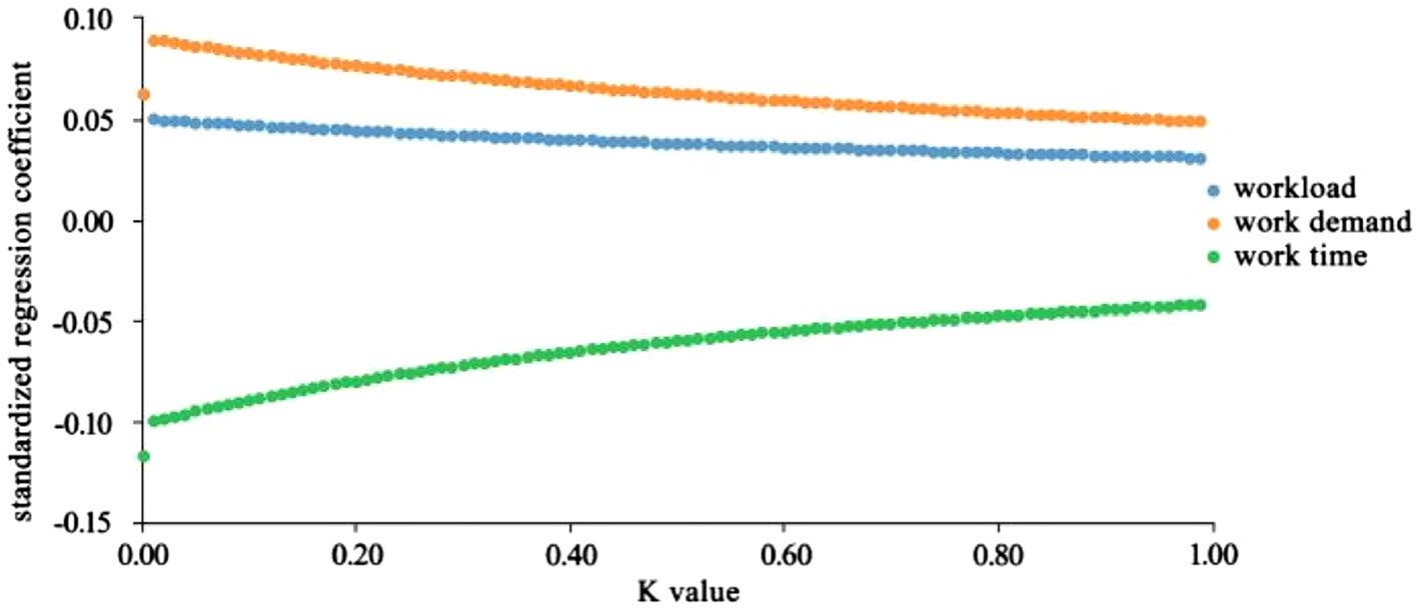
Ridge trace diagram of workload and its dimensions on miners’ unsafe state.

In this study, workload, work time and work demand were taken as independent variables, while miners’ unsafe state was taken as dependent variable. Ridge regression analysis was performed to obtain the ridge trace diagram, as shown in Fig. 1.

Figure 1 shows that when K is 0.99, the standardized regression coefficient of the independent variable tends to be stable. Therefore, 0.99 was taken as the K value for ridge regression analysis. The analysis results were shown in Table 7.

**Table 7:** Ridge regression analysis results of workload and its dimensions on miners’ unsafe state.

	Beta	t	p	R^2^	Adjust R^2^	F
Constant	–	21.485	0.000**	0.016	0.006	1.636 (p = 0.000)
Workload	0.051	1.675	0.004**			
Work time	0.042	1.532	0.043*			
Work demand	0.049	2.347	0.017*			

Beta represents standardized regression coefficient. t represents a test value for each independent variable one by one. p represents the significance value.

*Represents a significant level at p < 0.05.

**Represents a significant level at p < 0.01. R^2^ is the square of the multivariate correlation coefficient. F represents the analysis of variance test statistic.

The model passed the F test (f = 1.636, p = 0.000 < 0.05), indicating that at least one of workload, work time and work demand had an impact on miners’ unsafe state. Regression coefficient of workload was 0.051 (t = 1.675, p = 0.004 < 0.01), indicating that workload had a significant positive impact on miners’ unsafe state. Therefore, we established the validity of hypothesis H1. Regression coefficient of work time was 0.042 (t = 1.532, p = 0.043 < 0.05), indicating that work time had a significant positive impact on miners’ unsafe state. Therefore, we established the validity of hypothesis H1a. Regression coefficient of work demand was 0.049 (t = 2.347, p = 0.017 < 0.05), implying that work demand had a significant positive impact on miners’ unsafe state. Therefore, we established the validity of hypothesis H1b.

### Test of the moderating effect of job satisfaction

Regression analysis results of the moderating effect of job satisfaction on workload and miners’ unsafe state were shown in Table 8.

**Table 8:** The results of the moderating effect of job satisfaction on workload and miners’ unsafe state.

Variable	Model 1	Model 2	Model 3
Constant	2.771 (67.588**)	2.771 (67.497**)	2.756 (67.962**)
Workload	0.089(1.528)	0.085 (1.453)	0.134 (2.261*)
Job satisfaction		− 0.027 (− 0.420)	− 0.048 (− 0.756)
Workload*Job satisfaction			− 0.233 (− 3.460**)
Sample size	307	307	307
R^2^	0.008	0.008	0.046
Adjust R^2^	0.004	0.002	0.036
F value	2.334(p = 0.028)	1.252(p = 0.287)	4.855(p = 0.003)
△R^2^	0.008	0.001	0.038
△F value	2.334(p = 0.028)	0.176(p = 0.675)	11.972(p = 0.001)

R^2^ is the square of multivariate correlation coefficient. F represents the analysis of variance test statistic. p represents the significance value.

*Represents a significant level at p < 0.05.

**Represents a significant level at p < 0.01.

**Figure 2:**
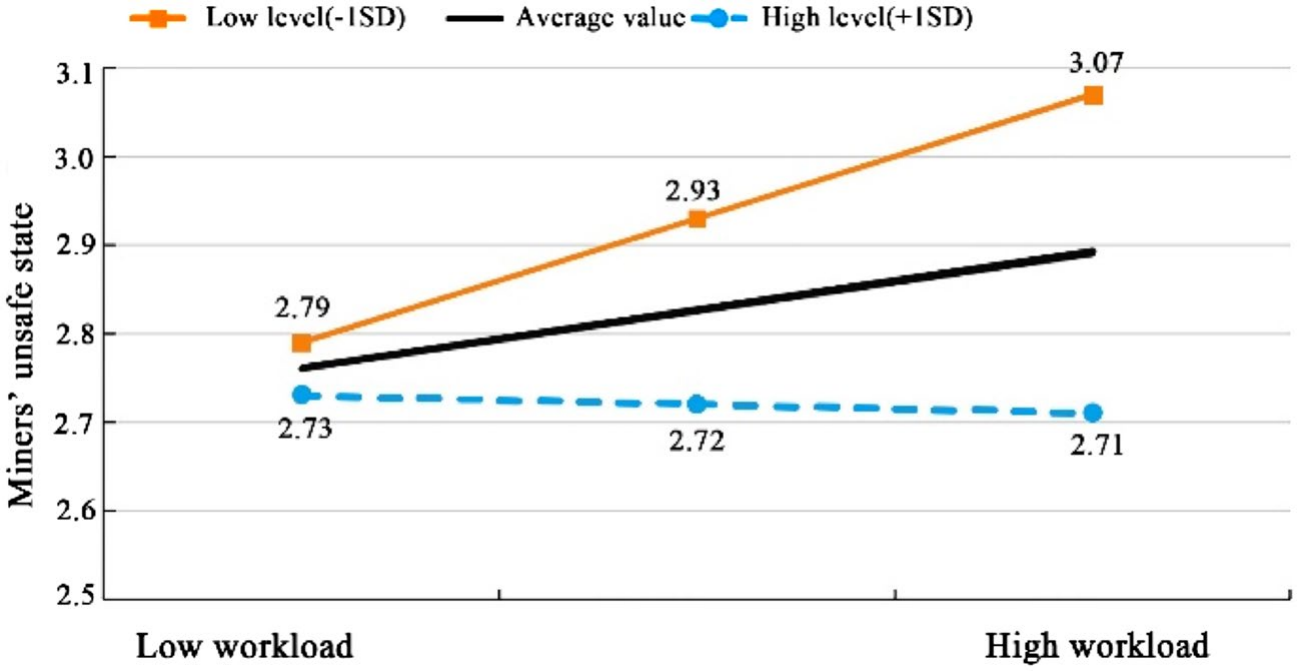
Simple slope diagram of the moderating effect of job satisfaction.

Table 8 shows that the test for moderating effect was divided into three models. The independent variable (workload) was included in Model 1. Model 2 adds a moderating variable (job satisfaction) on the basis of model 1. Model 3 adds an interaction term (the product term of the independent variable and the moderating variable) on the basis of model 2. The interaction between workload and job satisfaction was significant (t = − 3.460, p = 0.001 < 0.05). It means that when the workload has an impact on miners’ unsafe state, the magnitude of the impact is significantly different when the moderating variable (job satisfaction) is at different levels. To make the moderating effect of job satisfaction more intuitive, we analyzed the slope of job satisfaction when it plays a moderating effect. The results were shown in Table 9. The ordinate value was set to start from 2.5, and a simple slope diagram was drawn, as shown in Fig. 2.

**Table 9:** Results from simple slope analysis of the moderating effect of job satisfaction.

Moderating variable level	Regression coefficient	Standard error	t	p	95% Confidence interval	
Average value	0.134	0.059	2.261	0.024	0.018	0.251
High level (+ 1SD)	− 0.017	0.065	− 0.267	0.790	− 0.144	0.110
Low level(− 1SD)	0.286	0.082	3.496	0.001	0.126	0.446

Table 9 and Fig. 2 show that when the moderating variable of job satisfaction was at different levels (average level, high level (average plus 1 SD), low level (average minus 1 SD)), the independent variable of workload had a significant difference on the impact of the dependent variable of miners’ unsafe state. Compared to the low level of job satisfaction, in the case of high level of job satisfaction, there was a weak negative correlation between workload and miners’ unsafe state. When job satisfaction of miner reaches a higher level, then, as workload increases, miners’ unsafe state is less likely to appear, that is, the specific moderating effect. Job satisfaction can reduce the positive impact of workload on miners’ unsafe state. Thus, we established the validity of hypothesis H2.

Through the above empirical analysis, the results of the research hypotheses proposed in this study were summarized in Table 10.

**Table 10:** Judgment results of hypothesis test.

Assumed serial number	Research hypothesis	Is it established?
H1	Workload has a significant positive effect on miners’ unsafe state	Yes
H1a	Work time has a significant positive effect on miners’ unsafe state	Yes
H1b	Work demand has a significant positive effect on miners’ unsafe state	Yes
H2	Job satisfaction plays a moderating effect between workload and miners’ unsafe state	Yes

## Discussion

### The relationship between workload and miners’ unsafe state

We verified H1, H1a and H1b, indicating that workload, work time and work demand had significant positive impacts on miners’ unsafe state. Combining the theoretical knowledge of neuroscience, physiology and psychology, we can have a better understanding of complex stress-related problems. At the same time, we can effectively solve the research problems of behavioral science by using relevant means. The brain’s response to stress will cause anxiety and emotion in the human body, which will hinder the occurrence of safe behaviors, and then cause irreversible physiological and psychological damage. Haines et al.^42^ reported that increased work time leads to negative emotions among employees, and induces the occurrence of unsafe state. Working for a long time will lead to psychological stress and even illness in employees. McEwen^44^ documented that the occurrence of unsafe state such as increased blood pressure and heart rate is closely related to workload. Long-time work state and exhaustion of psychological resources required to meet work requirements reduces the ability of miners to cope with work. Moreover, physical fatigue and mental slack are likely to lead to the occurrence of miners’ unsafe state by affecting controllability of their behavior, leading to unsafe behavior, and eventually, to unsafe accidents.

Long time high-load work seriously damages the physical and mental health of miners, and reduces the ability of risk perception for miners. Therefore, coal enterprises should control the key areas associated with high incidences of coal mine accidents, and try to reduce or avoid the work of miners in this areas. Miners should be provided with sufficient rest time to ensure that they can achieve the basic requirements of high quality and efficient work. Moreover, the work time of all miners should not be more than 8 h. At the same time, miners with high labor intensities should be given necessary nutritional supplements and other economic compensation. Various recreational activities and collective learning, such as quality development training, badminton and other ball games, safety knowledge competition, among others should be encouraged. Not only can miners achieve the effect of relaxation and rest, they can also learn and consolidate the relevant safety knowledge.

### The moderating effect of job satisfaction in the relationship between workload and miners’ unsafe state

The impact of workload on miners’ unsafe state was verified. Job satisfaction was added into the model as a moderating variable, and fitting results verified H2. Job satisfaction had a moderating effect, and the moderating effect was significantly different at different levels. Combined with the research results of Chen et al.^49^, it is feasible to apply relevant variables to the field of coal mine safety, and the research results have certain similarities. Medical workers and miners are both groups with a large workload, and the quality of job satisfaction can directly affect the state of people. Verification of the moderating model of job satisfaction revealed that when miners are at a high level of job satisfaction, increased workload has less impact on their unsafe state.

Through functional near-infrared spectroscopy technology, the cognitive safety state of operators with different workload in harsh environment can be detected. When the workload level is relatively low, the cognitive signals in the brain carry out task loading, thus giving instructions to make the human body in a safe state^58^. And research shows that satisfaction levels are lower when the workload is heavier. There was a negative correlation between workload and job satisfaction^59^. When the working hours are extended and the employees cannot get proper rest, the job satisfaction will be significantly reduced^60^. Thus, improving job satisfaction can moderate the impact of workload on personnel insecurity. Job satisfaction is a kind of work attitude. As mining is a special occupation, it affects exertion of miners’ work enthusiasm, their own work life quality and safety awareness.

According to the hierarchy of needs theory, when miners have a high degree of job satisfaction, it means that they generally like the work they are engaged in. Job satisfaction can improve job performance through organizational commitment. Employees with less workload have more perfect work motivation, and it is easy to achieve the accumulation of their own work performance through organizational commitment^61^. They are concerned about their work and are willing to devote themselves to mining industry. At the same time, they have a high evaluation of their work and a positive attitude. When economic and emotional needs of miners are satisfied, increased workload may stimulate the enthusiasm of miners. As a result, they gain resource demands and recognition from their work, thereby minimizing the occurrence of unsafe state^62^. Overall outcomes include maintenance of a good work state, positive attitude, high work efficiency, low absence rates, and strong safety awareness. They have a sense of dependence, achievement and pride on the enterprise, and are willing to stay in the mine for a long time. When miners are not satisfied with their work, increased workload enhances the occurrence of miners’ unsafe state. If miners’ needs are not met, their dependence on work will be reduced. With increased workload, subjective feelings and fatigue of miners increases, chances of making mistakes will be greatly increased, resulting in an unsafe state. Therefore, job satisfaction levels for miners affects productivity and absenteeism rate of mining enterprises by affecting the state of miners, which plays a vital role in mine safety production.

## Conclusions

Based on the questionnaire survey and analysis of front-line employees of two coal mines in China, a model of the relationship between workload, job satisfaction and miners’ unsafe state was constructed, and the mechanism of the influence of workload on miners’ unsafe state was explored. The following conclusions are drawn.

i. Dividing workload into two dimensions of work time and work demand for research can better reveal the impact mechanism of workload on miners' unsafe state. The revised scale can be used to evaluate the miners' unsafe state. Developing more specific and detailed questionnaires requires additional research. In the future, more sensitive and detailed questions will be raised in combination with the experience in coal mine safety management.
ii. There is a significant correlation between workload, work time, work demand and miners’ unsafe state. Moreover, workload, work time and work demand have a positive impact on miners’ unsafe state. From analysis, it is only from the perspective of miners that a reasonable arrangement of work time and spiritual needs of supply can reduce the probability of occurrence of miners’ unsafe state.
iii. Job satisfaction plays a moderating effect in the relationship between workload and miners’ unsafe state. Compared to low job satisfaction levels, high job satisfaction has a greater moderating effect. Higher job satisfaction can adjust the workload of miners, so as to reduce the occurrence of miners’ unsafe state.

We provide strong empirical evidence and conceptual models that can play an important role in improving sustainable operation and management, reducing accident rate and making decisions on safety related employee appointment.

## Suggestions

Therefore, coal enterprises should pay more attention to changes in workload of miners and adopt corresponding measures to change their degree of satisfaction with their work. Based on the above conclusions, we put forward the following suggestions for improving miners’ unsafe state.

- Regularly assess the workload of miners. Studies have shown that high workload limit the distribution of employees’ attention. To improve the accuracy of employees’ perception of risk factors in the environment, the workload should be appropriate^63^. According to the evaluation results, reasonable arrangement of miners’ working hours and tasks can not only keep miners in a safe state, but also reflect the humanization and refinement of coal mine safety management.
- Improve miners’ job satisfaction and cultivate intrinsically safe miners. Through measures such as career clarification, salary marketization, and scientific performance appraisal, a scientific and fair human resource management system will be established to enhance miners’ sense of fair distribution. Strengthen the support of safety environment and create a good working atmosphere. Strengthen safety training to enable miners to easily identify risks in the working environment and realize their own value by improving their skills.
